# Association between a genetic index for digital dermatitis resistance and the presence of digital dermatitis, heel horn erosion, and interdigital hyperplasia in Holstein cows

**DOI:** 10.3168/jds.2023-24136

**Published:** 2024-07

**Authors:** A. Anagnostopoulos, M. Barden, B.E. Griffiths, C. Bedford, M. Winters, B. Li, M. Coffey, A. Psifidi, G. Banos, G. Oikonomou

**Affiliations:** 1 Department of Livestock and One Health, Institute of Infection, Veterinary and Ecological Sciences, University of Liverpool, Leahurst Campus, Liverpool, CH64 7TE, United Kingdom; 2 Agriculture and Horticulture Development Board, Coventry, CV3 4PE, United Kingdom; 3 Animal and Veterinary Sciences, Scotland's Rural College, Roslin Institute Building, Easter Bush, Midlothian, EH25 9RG, United Kingdom; 4 Department of Clinical Science and Services, Royal Veterinary College, North Mymms, Hertfordshire, AL9 7TA, United Kingdom

**Keywords:** digital dermatitis, genetic index, resistance, lameness

## Abstract

Digital dermatitis (DD) is a polybacterial disease endemic to most UK dairy farms. It poses a major financial and welfare threat and is characterized by high incidence and recurrence rates. We aimed to investigate the association between the UK EBV for resistance to digital dermatitis, the digital dermatitis index (DDI), and the frequency of DD, heel horn erosion (HHE), and interdigital hyperplasia (IH) in a population of Holstein dairy cows. We enrolled and genotyped 2,352 cows from 4 farms in a prospective cohort study. Foot lesion records were recorded by veterinary surgeons for each animal at 4 time points during a production cycle, starting at approximately 2 mo before calving and ending in late lactation. Importantly, these records were not used in the calculation of the DDI. Lesion records were matched to the animal's own DDI (n = 2,101) and their sire's DDI (n = 1,812). Digital dermatitis index values in our study population ranged from −1.41 to +1.2 and were transformed to represent distance from the mean expressed in SD. The relationship between the DDI and the presence of DD was investigated using a logistic regression model, with farm, parity, and a farm-parity interaction fitted as covariates. A multivariable logistic regression model was fitted to evaluate the relationship between HHE and DDI with farm fitted as a covariate. Finally, a univariable logistic regression model with DDI as explanatory variable was used to investigate the relationship between IH and DDI. The odds ratio of an animal being affected by DD was 0.69 for 1 SD increase in the animal's DDI (95% CI = 0.63–0.76). The odds of HHE and IH were 0.69 (95% CI = 0.62–0.76) and 0.58 (95% CI = 0.49–0.68) respectively for 1 SD increase in DDI. The adjusted probability of DD was 32% (95% CI = 27–36%) for cows with mean DDI value of 0, while it was 24% (95% CI = 20–29%) in cows with a DDI value of +1. Sire DDI breeding values were standardized in the same way and then binned into terciles creating an ordinal variable representing bulls of high, medium, and low genetic merit for DD resistance. The daughters of low genetic merit bulls were at 2.05 (95% CI = 1.60–2.64), 1.96 (95% CI = 1.53–2.50), and 2.85 (95% CI = 1.64–5.16) times greater odds of being affected by DD, HHE, and IH, respectively, compared with the daughters of high genetic merit bulls. The results of this study highlight the potential of digital dermatitis genetic indexes to aid herd management of DD, and suggest that breeding for resistance to DD, alongside environmental and management control practices, could reduce the prevalence of the disease.

## Abstract (author highlights)

The list of standard abbreviations for JDS is available at adsa.org/jds-abbreviations-24. Nonstandard abbreviations are available in the Notes.

## INTRODUCTION

Digital dermatitis (**DD**) is endemic in the majority of UK dairy herds (Laven and Hunt, 2001; Barker et al., 2010) and is often the most prevalent foot lesion recorded on dairy farms worldwide (Manske et al., 2002; Cramer et al., 2008; Solano et al., 2016). It is a complex polybacterial (Caddey and De Buck, 2021) infectious disease characterized by slow recovery (Relun et al., 2012; Biemans et al., 2018) and high recurrence rates (Berry et al., 2012; Krull et al., 2016).

The chronicity of DD infections, alongside high herd and within-herd prevalence, makes the disease a serious welfare issue (Bruijnis et al., 2012), and for this reason, DD is ranked by UK dairy farmers at the top of the list of diseases threatening cattle welfare and production (AHDB and RHW, 2021). The average cost of a DD case has been estimated to range between $64 (Dolecheck et al., 2019) and $132 (Cha et al., 2010), arising from a drop in milk production (Warnick et al., 2001; Relun et al., 2013; Gomez et al., 2015b), reduced conception rates (Hernandez et al., 2001), an increase in days from calving to conception (Gomez et al., 2015b), the cost of treatment, additional labor cost, a high chance of reinfection, and an increase in cull rates (Dolecheck et al., 2019).

The genetic background of resistance to DD could be used to aid DD control. Sousa Junior et al. (2023) recently performed a large-scale GWAS and identified several SNPs associated with susceptibility to DD. The heritability of resistance to DD has been reported to range from 0.04 to 0.28 (van der Waaij et al., 2005; Schöpke et al., 2015; Heringstad et al., 2018; Schneider et al., 2023). These relatively low heritability values do not indicate that breeding strategies for DD resistance would be unsuccessful because the genetic variance is sufficiently high allowing identification of genetically superior animals. Fertility, another “low heritability trait,” had a downward genetic trend that has been reversed over the last decade with the inclusion of the trait in genetic selection indexes (Cole and VanRaden, 2018).

Breeding for DD resistance, or resistance to other foot lesions, can be based on either direct or indirect traits. Indirect body conformation traits, such as legs and feet (**LF**), foot angle, and locomotion score (**LOC**), have been reported to be genetically correlated with foot lesion incidence (van der Waaij et al., 2005; Onyiro et al., 2008) and have been used for genetic selection to reduce lameness. The fact that such conformation traits are already collected by breed societies and are available for young animals (which are less likely to be affected by some of the foot lesions of interest) are some of the reported advantages justifying the use of indirect traits (Mcdaniel, 1997). Several countries, including Norway, Finland, Sweden, Denmark, Germany, Austria, and Canada (Egger-Danner et al., 2015; Malchiodi et al., 2018), compile foot lesion records in national databases, which are used to create direct genetic indexes for resistance to lameness. Foot-trimmer records are considered better quality phenotypes for such evaluations (Koenig et al., 2005; Heringstad et al., 2018), but farm records have also been used (Pritchard et al., 2013; Parker Gaddis et al., 2014). Underreporting of foot lesion events by farmers and the lack of a standardized system of diagnosis can decrease the quality of such phenotypic records.

Three sources of phenotypic data are used for the calculation of the lameness advantage index (**LAI**), published by the UK Agricultural and Horticultural Development Board (**AHDB**). The first source of information comes in the form of conformation data from the type classification scheme recorded by Holstein UK. Presence of DD in primiparous animals recorded by Holstein UK classifiers is the second source of phenotypic information. This screening takes place by visually examining the skin between the heel bulbs while the cows are standing and takes place once a year for every pedigree herd in the UK. The third source of information comes from lameness events recorded by farmers and reported to UK milk recording organizations (Cattle Information Services and National Milk Records). A stand-alone digital dermatitis index (**DDI**) was also made available by AHDB (2020). The DDI is calculated from DD records provided by Holstein UK classifiers and is expressed on a −2 to +2 scale, with positive values being favorable (AHDB, 2020).

We recently reported that cows with higher LAI breeding values had lower odds of sole ulcer (**SU**), sole hemorrhage (**SH**), and lameness (Barden et al., 2022). However, the associations between DDI and the incidence of DD, heel horn erosion (**HHE**), and interdigital hyperplasia (**IH**) have not yet been investigated. These foot lesions may share common causative mechanisms, as positive phenotypic and genetic correlations between them have been reported (van der Waaij et al., 2005; Malchiodi et al., 2017; Heringstad et al., 2018). Manske et al. (2002) reported a strong positive correlation between DD and HHE; IH was also strongly associated with DD and HHE in the same study. Holzhauer et al. (2008), monitored a population of 138 Holstein cows for a month and reported coinfection with DD in all IH cases and that HHE doubled the risk of DD. Finally, the impact of DD on claw conformation and HHE was investigated by Gomez et al. (2015a) in a population of 644 Holstein heifers screened 3 times over a period of 6 mo. The active ulcerative stage of DD increased the incidence and severity of HHE, the depth of the interdigital space and the accumulation of debris. Even if a causative relationship between these lesions cannot be definitively established, a common etiology is probable (Manske et al., 2002; Knappe-Poindecker et al., 2013).

Our objective was to study the association between DDI and the observed DD frequency in a cohort of genotyped cows with detailed foot lesion records that were not part of the reference population. Because DD has been associated with HHE and IH, the scope of our study was expanded to include these lesions.

## MATERIALS AND METHODS

This study was conducted following ethical approval from the University of Liverpool Veterinary Research Ethics Committee (VREC269a, VREC466ab).

### Farm Selection and Data Collection

Data analyzed here were collected as part of a large-scale prospective cohort study that has already been described in previous publications (Anagnostopoulos et al., 2021; Barden et al., 2022). Briefly, 4 commercial dairy farms were selected in North West England and North Wales based on distance and ease of arranging frequent visits for data collection. Farms A, B, and C housed a population of ∼180, ∼2,000, and ∼750 cows all year round and milked 3 times per day. Both formalin and copper sulfate solutions were used in footbaths on farms A, B, and C, and footbath frequencies were 3 times per week, twice a day, and once a day, respectively. Farm D milked a herd of ∼340 cows twice a day. The high-yielding group was housed all year round, while the low-yielding group grazed during the summer. Only formalin solutions were used in footbaths on farm D, and footbath frequency was 3 times per week. Milking cows on all farms were routinely trimmed at least twice a year, once before entering the dry period and once during the early lactation.

All animals from those 4 farms entering the last 2 mo of gestation were eligible for enrollment without any preselection taking place. A population of 2,352 Holstein cows and heifers across the 4 farms was enrolled in the study from February to October 2019. Animals were assessed at 4 time points: approximately 2 mo before the expected calving date (enrollment), 1 wk postcalving, during early lactation (∼80 DIM), and late lactation (∼200 DIM). Data were collected by qualified veterinary surgeons from February 2019 to July 2020. Blood samples were collected from each animal at enrollment and were used for genotyping and breeding value estimations. At each assessment point, cows were restrained in a foot trimming crush and received either a functional or therapeutic foot trim or a mild investigative trim, depending on the farm and time point, to record foot lesion data. More than 90% of the claw horn disruption and infectious lesion records were collected by a single researcher to ensure a better standardized case diagnosis. We recorded DD, HHE, and IH lesions according to the *ICAR Claw Health Atlas* (Egger-Danner et al., 2014). All cows were genotyped and the DDI GEBV for cows and their sires were provided by AHDB in the form of predicted transmitted abilities. Thirty-nine genetic indexes were available in total for our population after the August 2021 national evaluation (Barden et al., 2022). Foot lesion phenotypes collected during the study were not submitted to AHDB allowing for GEBVs produced by AHDB to be independent from our scores.

### Statistical Analysis

Digital dermatitis records from all feet of each cow assessed across the 4 time points were transformed into a binary variable (DD binary), where 0 represented nonaffected and 1 represented affected animals. Nonaffected animals had no DD lesions recorded on any foot at any of the assessment time points, whereas animals that had at least one DD lesion of any grade or severity on any foot throughout the study were regarded as affected. The same binary transformation was then repeated for HHE and IH lesions to create the observed lesion presence variables HHE binary and IH binary, respectively.

Disease records for each animal were then merged with the cows' published DDI GEBVs and their sire's GEBVs. Only animals with at least one foot lesion record and available GEVB were included in final analyses. Parity was transformed into a binary variable, with 1 and 2 representing primiparous and multiparous animals, respectively. Data handling and statistical analysis were performed in R (4.0.1; R Core Team, 2021) using RStudio (RStudio Team, 2020).

The GEBVs for DDI, sire DDI, and the remaining 38 available indexes were standardized by subtracting their respective mean and dividing by their respective SD. This allowed for any potential correlation with lesion presence to be comparable between indexes of originally nonequal units. Finally, the sire DDI was binned into terciles representing bulls of low, medium, and high genetic merit for resistance to DD.

Univariable models were fitted to investigate the association of DD binary, HHE binary, and IH binary with cows' DDI, parity, and farm before fitting multivariable models. Log-linearity was evaluated by plotting the logit probability against the DDI for these univariable models. Estimated marginal means were plotted using the *emmeans* package (Lenth et al., 2020) to detect interactions between the explanatory variables. The final logistic regression model with DD binary as a dependent variable included the cows' DDI, parity, farm, and farm × parity interaction as explanatory variables. For HHE binary, cows' DDI and farm were kept in the final model. For IH binary only cows' DDI was kept as an explanatory variable in the final model. The performance of the logistic regression models were assessed using the *performance* package (Lüdecke et al., 2021), testing for collinearity between explanatory variables (where appropriate) and creating binned residual plots, while model fit was evaluated using Hosmer-Lemeshow tests. Model-adjusted probabilities for DD, IH, and HHE lesion outcomes based on different values of DDI were calculated using the *ggeffects* package (Lüdecke, 2018). Predicted probabilities for DD were calculated for each farm separately correcting for the average effect of parity and then again for each parity group separately correcting for the average effect of farm.

The 3 final logistic regression models were fit again but this time using the sire DDI as an explanatory variable (instead of the cows' DDI). Binned residual plots and Hosmer-Lemeshow tests were run again to evaluate the sire models' fit. Tukey's tests comparing the odds of DD, HHE, and IH between the 3 sire DDI groups were run using the *emmeans* package (Lenth et al., 2020).

Finally, after evaluating the association of DDI and presence of DD, we also tested other traits that have been historically linked to foot health. We fit our final DD model again replacing DDI with LOC, LF, rear leg side view (**RLSV**), and LAI each time. For posterity, we had to test all 39 available genomic traits in this way and not arbitrarily select some. This multiple testing increased the chance of type 1 error and as a result, *P-*values were adjusted using the Bonferroni multiple-comparison correction.

## RESULTS

From the originally enrolled 2,353 animals, 252 were missing either foot records or GEBVs; 2,101 cows had at least one foot lesion record and could be matched to published DDI GEBVs. Sire DDI GEBVs were available for 1,812 of these cows. More than 95% of those 2,101 animals had at least 2 assessments with foot lesion records. The distribution of cows within farm, parity, and DDI groups as well as the presence of each lesion during the study period for each one of these groups are summarized in Table 1.

**Table 1:** Lesion presence during the study period within each cow digital dermatitis index (DDI), parity, and farm group^1^

Item	N	DD		HHE		IH	
		n	prev. (%)	n	prev. (%)	n	prev. (%)
DDI^2^							
≤−1.5	172	88	51.2	137	79.7	28	16.3
>−1.5 ≤ −0.5	396	164	41.4	284	71.7	35	8.8
>−0.5 ≤ 0.5	866	334	38.6	547	63.2	44	5.1
> 0.5 ≤ 1.5	558	176	31.5	321	57.5	18	3.2
>1.5	109	24	22.0	45	41.3	4	3.7
Sum	2,101	786	37.4	1,334	63.5	129	6.1
Parity^3^							
1	583	270	46.3	360	61.7	36	6.2
2	1,518	516	34.0	974	64.2	93	6.1
Sum	2,101	786	37.4	1,334	63.5	129	6.1
Farm							
A	81	33	40.7	62	76.5	8	9.9
B	1,402	542	38.7	938	66.9	83	5.9
C	406	118	29.1	201	49.5	20	4.9
D	212	93	43.9	133	62.7	18	8.5
Sum	2,101	786	37.4	1,334	63.5	129	6.1

1 DD = digital dermatitis; HHE = heel horn erosion; IH = interdigital hyperplasia; N = number of cows per group; n = number of cows with a lesion; prev. (%) = percentage of cows with a lesion at least once throughout the study within each group.

2 Binned cow DDI breeding values expressed in SD from the study mean (high values are desirable).

3 Parity groups with 1 representing primiparous and 2 representing multiparous animals.

The logistic regression models, which aimed to investigate the relationship between DDI and lesion presence, did not violate any assumptions of log-linearity, collinearity between explanatory variables, and residual distribution and passed the Hosmer-Lemeshow test of model fit. The explanatory power of the models was low with Tjur's R^2^ of 0.09, 0.05, 0.02 for models with DD, HHE, and IH as an outcome, respectively. The results of these logistic regression models are presented in Table 2. For each 1-point or SD increase in DDI, the odds ratios (**OR**) were 0.69 (95% CI = 0.63–0.76), 0.39 (95% CI = 0.62–0.76), and 0.58 (95% CI = 0.49–0.68) for DD, HHE, and IH, respectively.

**Table 2:** Multivariable logistic regression model OR with lesion presence in the studied cows as the outcome and parity, farm, and animal's own digital dermatitis index (DDI) breeding values as explanatory variables^1^

Item	DD			HHE			IH		
	OR	95% CI	*P*-value	OR	95% CI	*P*-value	OR	95% CI	*P*-value
DDI^2^	0.69	0.63–0.76	<0.001	0.69	0.62–0.76	<0.001	0.58	0.49–0.68	<0.001
Farm									
A	Referent								
B	2.98	1.37–6.89	0.007	0.62	0.35–1.04	0.080			
C	0.47	0.17–1.28	0.133	0.30	0.17–0.51	<0.001			
D	0.19	0.04–0.69	0.019	0.57	0.31–1.02	0.063			
Parity^3^									
1	Referent								
2	1.68	0.65–4.49	0.289						
Interaction^4^									
Parity 2: farm A	Referent								
Parity 2: farm B	0.18	0.06–0.47	0.001						
Parity 2: farm C	1.22	0.38–3.95	0.739						
Parity 2: farm D	8.51	1.96–46.4	0.007						

1 DD **=**digital dermatitis; HHE = heel horn erosion; IH = interdigital hyperplasia. The binary lesion presence DD binary, HHE binary, and IH binary, respectively, used as the dependent variable of the logistic regression models. The intercepts (SE) for the DD, HHE, and IH models were 0.47 (0.4), 3.3 (0.27), and 0.06 (0.1), respectively.

2 Standardized breeding values for the DD genetic index expressed in SD from the mean.

3 Parity group with 1 representing primiparous and 2 representing multiparous animals.

4 Interaction between farm and parity fitted as explanatory variable.

**Figure 1:**
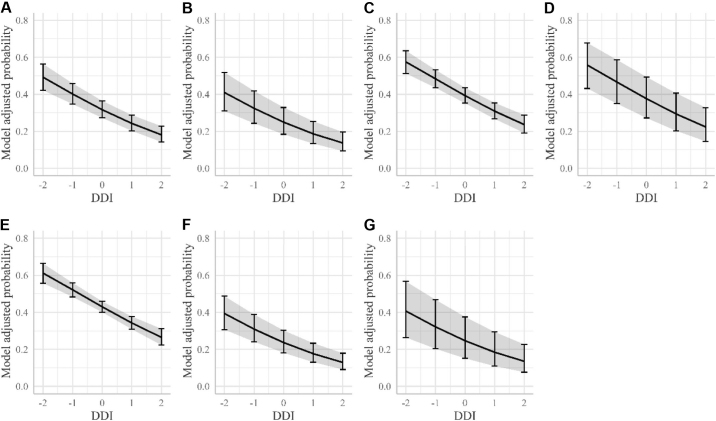
Model-adjusted probabilities of digital dermatitis (DD) development. The adjusted probability of presence of digital dermatitis is plotted against the animals' own digital dermatitis index (DDI) breeding values expressed in SD from the mean (continuous line). The bars represent the 95% CI for the adjusted probability of DD for cows with a DDI of −2, −1, 0, 1, and 2. (A) Model-adjusted probabilities corrected for the effect of farm and parity. Model-adjusted probabilities for parity groups 1 (B) and 2 (C), corrected for the effect of farm. Model-adjusted probabilities for farms A, B, C, and D (D–G, respectively), corrected for the effect of parity.

**Figure 2:**
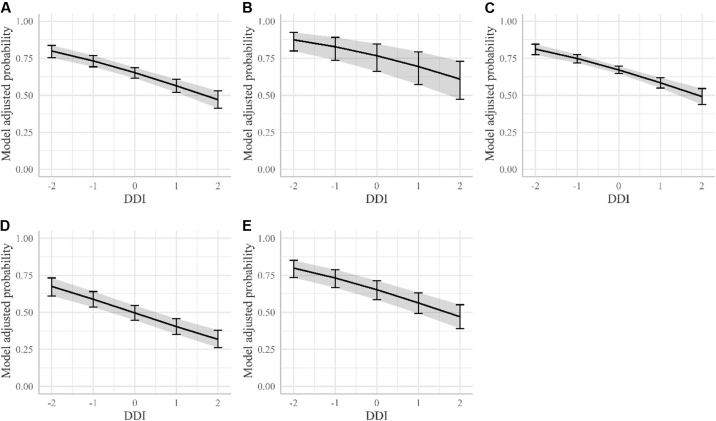
Model-adjusted probabilities of heel horn erosion (HHE) development. The adjusted probability of presence of HHE is plotted against the animals' own digital dermatitis index (DDI) breeding values expressed in SD from the mean (continuous line). The bars represent the 95% CI for the adjusted probability of HHE for cows with a DDI of −2, −1, 0, 1, and 2. (A) Model-adjusted probabilities corrected for the effect of farm. Model-adjusted probabilities for farms A, B, C, and D (B–E, respectively).

**Figure 3:**
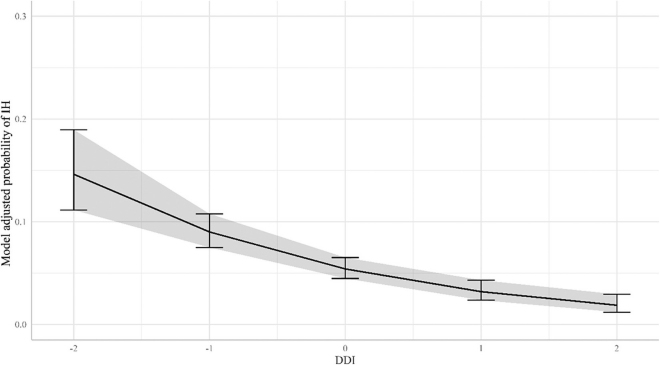
Model-adjusted probabilities of interdigital hyperplasia (IH) development. The adjusted probability of presence of IH is plotted against the animals' own digital dermatitis index (DDI) breeding values expressed in SD from the mean (continuous line). The bars represent the 95% CI for the adjusted probability of IH for cows with a DDI of −2, −1, 0, 1, and 2.

Model-adjusted probabilities were plotted against the animals' DDI values in Figures 1, 2, and 3 for DD, HHE, and IH, respectively. These plots also display the DD predicted probability for each farm separately (corrected for the average effect of parity) and for each parity group separately (corrected for the average effect of farm). A decrease in DD predicted probability as DDI values increased was observed regardless of farm or parity group.

The results of the multivariable logistic regression models investigating the relationship between sire DDI breeding values and lesion presence are summarized in Table 3. Animals sired by bulls of low and medium genetic merit for DDI were at 2.05 (95% CI = 1.60–2.64) and 1.67 (95% CI = 1.28–2.16) times greater odds of being affected by DD during our study compared with animas sired by bulls of high genetic merit for DDI. Model-adjusted probability of DD for the daughters of high, medium, and low genetic merit bulls were 20.3% (95% CI = 15.3–26.4%), 29.8% (95% CI = 23–37.6%), and 34.3% (95% CI = 27.2–42.1%), respectively, corrected for the effect of farm and parity. Animals sired by bulls of low and medium genetic merit were at 1.96 (95% CI = 1.53–2.5) and 1.5 (95% CI = 1.18–1.9) greater odds of being affected by HHE respectively and 2.85 (95% CI = 1.64–5.16) and 2.45 (95% CI = 1.4–4.48) greater odds of being affected by IH. The difference in the odds of lesion presence between animals sired by bulls of low and medium genetic merit was not significant for any of the 3 lesions evaluated.

**Table 3:** Multivariable logistic regression model OR with lesion presence in the studied cows as the outcome and parity, farm, and cow sire's digital dermatitis index (DDI) breeding values as explanatory variables^1^

Item	DD			HHE			IH		
	OR	95% CI	*P*-value	OR	95% CI	*P*-value	OR	95% CI	*P*-value
Sire DDI^2^									
High	Referent								
Medium	1.67	1.28–2.16	<0.001	1.50	1.18–1.9	0.001	2.45	1.40–4.48	0.002
Low	2.05	1.60–2.64	<0.001	1.96	1.53–2.5	<0.001	2.85	1.64–5.16	<0.001
Farm									
A	Referent								
B	2.83	1.31–6.53	0.010	0.70	0.40–1.17	0.191			
C	0.41	0.11–1.37	0.159	0.33	0.18–0.57	<0.001			
D	0.09	0.01–0.55	0.029	0.45	0.24–0.84	0.014			
Parity^3^									
1	Referent								
2	1.84	0.72–4.89	0.212						
Interaction^4^									
Parity 2: farm A	Referent								
Parity 2: farm B	0.20	0.07–0.54	0.002						
Parity 2: farm C	1.33	0.34–5.61	0.686						
Parity 2: farm D	11.7	1.72–238	0.032						

1 DD **=** digital dermatitis; HHE = heel horn erosion; IH = interdigital hyperplasia. The binary lesion presence DD binary, HHE binary, and IH binary, respectively, used as the dependent variable of the logistic regression models. The intercepts (SE) for the DD, HHE, and IH models were 0.28 (0.4), 2.07 (0.28), and 0.03 (0.25), respectively.

2 Sire breeding values for the DD genetic index binned into terciles.

3 Parity group with 1 representing primiparous and 2 representing multiparous animals.

4 Interaction between farm and parity fitted as explanatory variable.

There was an association between LF, LOC, and LAI indexes and DD presence with OR of 0.73 (95% CI = 0.66–0.80), 0.74 (95% CI = 0.67–0.81), and 0.82 (95% CI = 0.74–0.90), respectively, for every 1 SD increase of the breeding value. From the remaining 34 genomic traits tested, only condition score and chest width were associated with DD presence after the Bonferroni correction, with an OR of 0.83 (95% CI = 0.75–0.91) and 0.85 (95% CI = 0.78–0.94), respectively. The OR, Bonferroni-corrected, and unadjusted *P*-values for all 39 traits tested are available as Supplemental Material (see Notes).

## DISCUSSION

Our main goal was to evaluate the association between the DDI and the frequency of DD lesions in our study population. This analysis was further extended to include the association between DDI and HHE and IH. Lesion records collected for this study were not used in the calculation of the GEBVs.

We found that 1 SD increase in the animal's own DDI breeding value results in a 31% reduction in the odds of DD (OR = 0.69, 95% CI = 0.63–0.76) in our population. For context, the most recent meta-analysis on the efficacy of footbaths on DD prevention (Jacobs et al., 2019) showed that the effect of the industry's most commonly used compounds CuSO_4_ and formalin did not differ from no foot bathing and only achieved a trend in DD odds reduction of 56% (OR = 0.44, 95% CI = 0.10–1.70) and 47% (OR = 0.53, 95% CI = 0.07–3.83) respectively. Improving the population average DDI by just 1 SD could result in a significant reduction in DD frequency, further supporting the idea that although foot health is affected by management practices (such as footbaths), breeding for lesion resistance can further aid long-term improvements in foot health (van der Linde et al., 2010).

The animals' own DDI breeding values in our study were strongly associated with the observed DD presence suggesting that female genetic selection based on their GEBVs is possible. Dhakal et al. (2015) reported that farm records of infectious foot lesions combined with genomic data resulted in an increase of 0.24 in the reliability of the estimated breeding values compared with pedigree data. Studies on the genomic evaluation of foot health (Malchiodi et al., 2020) and DD specifically (Malchiodi et al., 2018) using national foot-trimmer record databases have been published in Canada. According to a recent Canadian study (Malchiodi et al., 2020), the daughters of the top 10 bulls, regarding foot health breeding values, were free from DD in 95% of the foot trimming records, whereas the daughters of the bottom 10 were free from DD in only 64% of the records. Foot health improvement based on sire breeding value selection is further supported by our study. Animals sired by bulls of low and medium genetic merit regarding DD resistance were at 2.05 (95% CI = 1.60–2.64) and 1.67 (95% CI = 1.28–2.16) times greater odds of being affected by DD during our study.

The reduction in odds was similar to that of DD in the case of HHE (OR = 0.69, 95% CI = 0.62–0.76) and IH (OR = 0.58, 95% CI = 0.49–0.68). Studies investigating the genetic parameters of foot lesions often group DD and interdigital dermatitis (**ID**) together into a single dermatitis category. A strong positive genetic correlation between dermatitis (DD and ID) and HHE has been reported, ranging from 0.58 to 0.87 (Buch et al., 2011; Johansson et al., 2011; Ødegård et al., 2013). One study reported genetic correlation of 0.66 between IH and a trait that combined DD, ID, and HHE lesions (van der Spek et al., 2013). Studies that distinguished DD as a separate lesion reported genetic correlations with HHE, IH, and ID of 0.3, 0.11 to 0.65, and 0.44 to 0.88, respectively (van der Linde et al., 2010; Gernand et al., 2012; Malchiodi et al., 2020). Many epidemiological studies investigating potential risk factors for DD, hypothesize that DD, HHE, and IH arise from the same disease process and may share a common causative mechanism, or at the very least, the chronic irritation caused by DD increases the risk of IH development (Manske et al., 2002; Holzhauer et al., 2006, 2008; Solano et al., 2016) Therefore, it is not unexpected that the DDI is strongly associated with HHE and IH presence in our study.

The DDI is used, alongside other traits, in the calculation of the LAI, which is based on farmer-recorded lameness events of all etiologies, including DD. This explains the high correlation (0.69) between the 2 breeding values, reported in our previous publication (Barden et al., 2022). A positive correlation was also found previously between DDI and LF breeding values (0.45), as well as between DDI and LOC breeding values (0.47; Barden et al., 2022). Negative genetic correlations between DD susceptibility and LF is well documented, ranging from −0.27 to −0.63 (Koenig et al., 2005; van der Waaij et al., 2005; Onyiro et al., 2008; van der Linde et al., 2010), while a genetic correlation of −0.31 to −0.67 has been reported between LOC and DD (van der Waaij et al., 2005; Onyiro et al., 2008; van der Linde et al., 2010). The LAI, FL, and the LOC traits were all associated with lower odds of DD infection in our study, yet the effect size of DDI was greater. We found no substantial correlation between the DDI breeding values and the RLSV breeding values in our previous study (Barden et al., 2022). Most studies report either weak positive (0.13; Uggla et al., 2008) or no correlation between RLSV and DD (van der Waaij et al., 2005; Onyiro et al., 2008; van der Linde et al., 2010). The RLSV has also been reported to not affect overall foot health (Häggman et al., 2013) or the incidence of infectious foot lesions (Laursen et al., 2009; Ødegård et al., 2014). The RLSV was not associated with the presence of DD in our study, after the application of the Bonferroni correction.

The association between low BCS and lameness is relatively well established by now. Many studies have shown that cows with BCS lower than 2.5 are not only more likely to be lame (Espejo et al., 2006; Dippel et al., 2009; Hoedemaker et al., 2009) but are also less likely to recover from a lameness event (Lim et al., 2015). It has been suggested that low BCS cows, having undergone a period of fat mobilization, lose some of the adipose tissue of the digital cushion and are more likely develop a lameness causing lesion (Bicalho et al., 2009). Debating whether a lameness event results in a lower BCS or a drop in BCS is a risk factor for lameness causing lesions is beyond the scope of this article. However, Green et al. (2014) reported the results of a longitudinal study spanning 44 mo, in which cows with BCS lower than 2.5 were more likely to develop a SU, SH, or white line lesion in the next 2 mo, but that was not the case for DD lesions. In our study, the OR of DD presence for 1 SD increase in the condition score breeding values was 0.83 (95% CI = 0.75–0.91). Potentially, cows that are genetically better at retaining higher BCS, cope better during periods of metabolic or inflammatory stress and as a result are less likely to be infected with DD. Supporting this hypothesis, early lactation heifers that dropped to BCS bellow 2.5 were more likely to be infected by DD in a study by Schöpke et al. (2013). This could also explain why the OR of DD was 0.85 (95% CI = 0.78–0.94) for 1 SD increase in chest width breeding values, because genetic correlation of 0.72 between chest width and DMI has been reported for early lactation cows (Williams et al., 2022).

Overall, the DDI associated well with DD, HHE, and IH lesion presence and, according to our previous publication (Barden et al., 2022), does not have any undesired correlations with other production traits. This is in line with other studies underlining the importance of incorporating direct foot health traits in the creation of indexes (Koenig et al., 2005; Swalve et al., 2008; van der Linde et al., 2010) that can be used to select for lameness resistance. Future genetic or genomic (Dhakal et al., 2015) evaluations based on reliable foot lesion records will further increase the accuracy of these foot health traits (Heringstad et al., 2018) as the reference population expands.

Genetic evaluation data were available for 2,101 out of the 2,353 animals enrolled, and only 1,812 of those could be matched to sire genetic information. Failure of DNA extraction was the most common cause of missing genotypic data for enrolled animals, and we expect this to have happened at random. Despite using a robust detailed foot lesion dataset, an argument could be made that lesions might have been missed between assessments especially for animals with incomplete records. However, only a small fraction of the population had missing foot lesion records for more than one assessment. Additionally, the fact that a single lesion on a single foot during any assessment would result in animals being classified as affected further minimizes the impact of this limitation. We cannot claim that the 4 farms that participated in this study represent the full range of herd sizes and management practices that can be found in the UK. In addition to that, 67% of animals were enrolled on farm B. Correcting for the effect of parity, however, the same trends could be seen for all farms regarding DD presence. Our models achieved very low Tjur's R^2^ values (0.02–0.09) indicating a small explanatory power and predictive capacity of our models.

## CONCLUSIONS

The results of this study support that the DDI could be used to select animals with better genetic resistance to DD, HHE, and IH. We found a strong negative association between these lesions and the cows' own DDI breeding values. Daughters of bulls in the high DDI category were less likely to develop these lesions during our study, indicating that sire selection could be used for hoof-health genetic improvement. The limited discriminatory power of our models indicates the importance of environmental factors on foot lesion development and as a result, genetic selection should supplement and not replace good lameness management practices.
